# BRCA2 gene mutations in families with aggregations of breast and stomach cancers

**DOI:** 10.1038/sj.bjc.6600562

**Published:** 2002-10-07

**Authors:** A Jakubowska, K Nej, T Huzarski, R J Scott, J Lubiński

**Affiliations:** Department of Genetics and Pathology, Pomeranian Academy of Medicine, Sczcecin, Poland; Department of Molecular Biology, Inter-University Unit, University of Szczecin and Pomeranian Academy of Medicine, Szczecin, Poland; Discipline of Medical Genetics, University of Newcastle, Newcastle, Australia

**Keywords:** BRCA2 gene, mutation, stomach cancer, breast cancer

## Abstract

Stomach cancer ranks second to lung cancer in the global cancer burden. It is estimated that 25% of families meeting the criteria for hereditary diffuse gastric carcinoma (HDCG) will have germline mutations in the E-cadherin gene. Evidence suggests that stomach cancer might also be a malignant manifestation of other inherited predispositions to disease. Recently, it has been reported that the incidence of stomach cancer is significantly increased in BRCA2 gene mutation carriers. We analysed by direct sequencing the BRCA2 gene in 29 breast cancer patients derived from 29 families with an aggregation of at least one female breast cancer diagnosed before the age of 50 years and one male stomach cancer diagnosed before the age of 55 years. In all but one of these families at least one additional relative was also affected by a malignant tumour. We identified three frameshift mutations and three sequence variants – potentially missense mutations, in six unrelated patients representing 20.7% (six out of 29) of the families investigated. Our results confirm that BRCA2 gene mutations are also associated with familial aggregations of not only breast but also of stomach cancer. In comparison to the number of cancers expected in the study population compared to the general population there is an over-representation of several cancers with significant confidence intervals to suggest that the associations are real and not a selection artefact.

*British Journal of Cancer* (2002) **87**, 888–891. doi:10.1038/sj.bjc.6600562 www.bjcancer.com

© 2002 Cancer Research UK

## 

Stomach cancer is the second most common epithelial cancer, and thereby represents a significant global cancer burden (La Vecchia *et al*, 1992). The first description of a genetic predisposition to familial stomach cancer came from the identification of germline mutations of the E-cadherin gene in Maori kindreds from New Zealand, where patients presented with an early onset, diffuse type cancer (Becker *et al*, 1994; Guilford *et al*, 1998). It has been estimated that only 25% of families meeting the criteria for hereditary diffuse gastric carcinoma (HDCG) will have germline mutations in the E-cadherin gene (Caldas *et al*, 1999). Stomach cancer has also been shown to be part of the tumour spectrum in other inherited syndromes, including hereditary non-polyposis colon cancer, otherwise known as HNPCC (Scott *et al*, 2001a), familial adenomatous polyposis (FAP) (Scott *et al*, 2001b), Peutz–Jeghers syndrome (Hizawa *et al*, 1993), Cowden's syndrome (Hamby *et al*, 1995) and the Li–Fraumeni syndrome (Scott *et al*, 1993).

Recently it has been shown that not only breast and ovarian but also other cancers are over-represented in BRCA2 linked families (BCLC, 1999; Johannsson *et al*, 1999). It has also been shown that the frequency of the BRCA2 6174delT mutation among consecutive Jewish patients with stomach cancer is 5.7%, which is approximately five times higher than in the general population (Figer *et al*, 2001). There is also evidence to suggest that the increased frequency of stomach cancers in BRCA2 carriers may be sex related, as it occurs primarily in males (BCLC, 1999; Johannsson *et al*, 1999).

In Polish families, with a strong aggregation of breast and ovarian cancers and no other cancer, the frequency of BRCA2 mutations is very low and does not exceed 5–10% (van der Looij *et al*, 2000; Grzybowska *et al*, 2000; Górski unpublished data).

Given the high frequency of breast and stomach cancer families observed in our familial breast cancer data base, we analysed the BRCA2 gene by direct DNA sequencing to determine the frequency and nature of BRCA2 germline mutations in families where there was a clear aggregation of breast and male stomach cancers occurring at early ages.

## MATERIALS AND METHODS

Twenty-nine families with an aggregation of at least one female breast cancer diagnosed before the age of 50 years and one male stomach cancer diagnosed under the age of 55 years were available for study. In 28 of these families at least one additional relative was diagnosed with a malignant tumour (Table 1

**Table 1:**
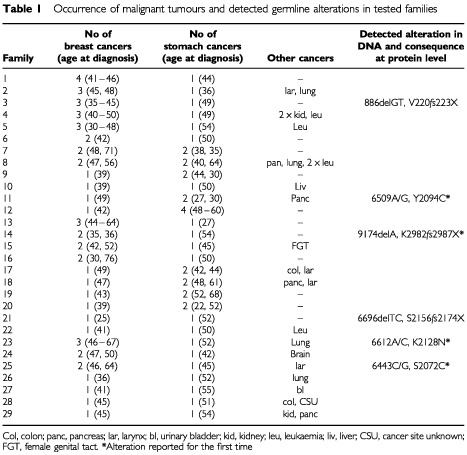
Occurrence of malignant tumours and detected germline alterations in tested families

). In 12 families stomach cancer was diagnosed in a first-degree relative of an early onset breast cancer proband (families 1–12). In 17 breast cancer families, stomach cancer was diagnosed among second degree relatives through an unaffected woman (10 cases, families 13–22) or through an unaffected male (seven cases, families 23–29). A control population of 100 healthy unaffected unrelated individuals was used for the assessment of potential pathogenic missense changes. For a comparison of the clinical features and age of diagnosis of malignancies of the 29 families see Table 1.

Peripheral blood for DNA isolation was taken from all index patients affected by breast cancer and from the 100 healthy controls (Lahiri and Schnabel, 1993).

None of the cancer patients showed signs of the five common Polish founder BRCA1 mutations (Górski *et al*, 2000). The entire coding sequence of the BRCA2 gene was amplified in 27 separate PCR reactions, using primers and conditions described previously (BCLC, http://www.nhgri.nih.gov/Intramural_research/Lab_transfer/Bic/Member/BRCA2.html), with minor modification. Instead of subdividing exons 11, 10, 14 and 27 due to their size, each were amplified in one fragment, using primers located in the adjacent introns. After purification, all PCR products were analysed on an ABI 377 DNA Sequencer with the primers described previously (BCLC,http://www.nhgri.nih.gov/Intramural_research/Lab_transfer/Bic/Member/BRCA2.html).

Standard incidence rates (SIRs) and confidence intervals (CIs) for the various cancer sites were calculated by the conventional method, which assumes all events are independent, and by a method which adjusts for intrafamilial correlation. The unadjusted method uses the formulae: SIR=O/E, SE(log SIR)=1/O^1/2^, so that a 95% CI for the estimated SIR is given by O/E×exp(±1.96×1/O^1/2^), where O is the observed number of cases and E is the expected number of cases according to the standard population rates. In our case, the standard rates are the Polish cancer incidence rates. The expected number of cases in each family is the standard rate times the number of family members. Expected number of cases for female-only cancers were calculated using the corresponding number of female family members.

The same results are obtained by fitting a Poisson model with only a constant term to the observed number of cases, using the corresponding expected number of cases in each family as the total exposure (or denominator of rate) for each family (see Clayton and Hills, 1993). This suggests an approach for adjusting for intrafamilial correlation, which is to fit the same model but to calculate robust standard errors that allow for this extra source of variation. The results for the adjusted approach are affected mainly via their standard errors, but if the number of observed cases is small, the SIR estimate will also be affected. All other statistical tests were performed using the STATA statistical package.

## RESULTS

From the 29 families, three frameshifts and three variants, potentially missense mutations, in six unrelated probands representing – 20.7% of families were identified. Two of the detected frameshift mutations (886delGT, 6696delTC) have been previously reported in BIC (http://www.nhgri.nih.gov/Intramural_research/Lab_transfer/Bic/index.html). One mutation (9174delA) localised in exon 22 and the three potential missense changes represented novel variations in the BRCA2 gene (Table 1). None of these changes were present in the 100 normal healthy control samples, consistent with the possibility that they are missense changes and are more than just rare polymorphisms.

**Figure 1:**
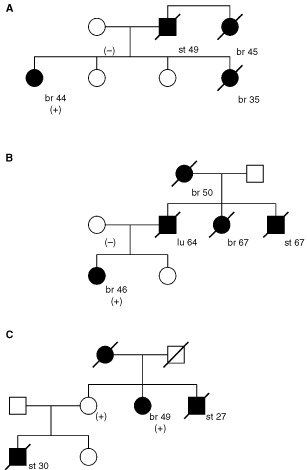
Pedigrees of three families carrying the BRCA2 alterations: 3 (A), 23 (B) and 11 (C). st, stomach cancer; br, breast cancer; lu, lung cancer; pan, pancreatic cancer; numbers in brackets indicate age at diagnosis of cancer; (+), (−) indicate presence or absence of BRCA2 alteration.

In three families the association of detected abnormalities in the BRCA2 gene with stomach cancer was supported by analyses of the occurrence of these changes in relatives. In families No 3 and 23 mutations were detected in probands with breast cancer but not in the maternal side of family where stomach cancer was not observed (Figure 1A,B

). In family No 11 the mutation was present in the proband and an obligatory carrier – the sister of proband being the mother of a stomach cancer patient (Figure 1C). DNA from relatives of the probands from families 14, 21 and 25 with BRCA2 gene alterations was unavailable for analysis.

In families where the occurrence of breast and stomach cancers was among 1° relatives, BRCA2 abnormalities were detected in two out of 12 cases (one frameshift, one assumed missense variation; 16.7%). In families with stomach cancers among 2° relatives, BRCA2 changes were identified in four out of 17 cases (two frameshifts, two assumed missense variations; 23.5%).

To further verify the uniqueness of the population under investigation, a statistical comparison was performed to determine if the observed cancers in the families were due to chance or most probably a result of a genetic predisposition. The relative frequency of the reported malignancies, compared to those of the general population, revealed several significant differences as shown in Table 2

**Table 2:**
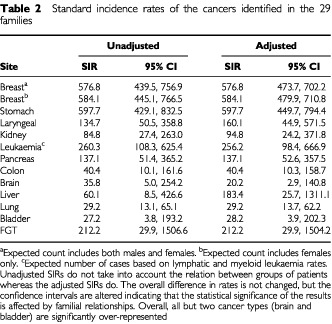
Standard incidence rates of the cancers identified in the 29 families

.

As expected in the study population breast and stomach cancers are over-represented with a high degree of confidence. Interestingly, leukaemia is also significantly over-represented followed by pancreatic cancer. All other cancers shown in Table 2 (except brain and bladder) are over-represented, but the confidence intervals were such that the significance was not as striking by virtue of the fact that the actual numbers of cancers within the study population were low.

When the families are sub-divided into those that harbour mutations in BRCA2 and compared to those that do not, further differences and similarities were observed. When compared against each other the overall frequency of cancers in the mutation negative group was significantly different (*P*<0.001) to that of the mutation positive group (25.3 and 17.5%, respectively). The frequency of breast cancer within the mutation negative group accounted for the difference in cancer incidence between the two groups. The frequency of stomach cancer was identical in both groups (7%). There were too few other cancers to determine if there were differences between the two groups.

Finally, a comparison was made between the frequency of BRCA2 mutations in this study population compared to the frequency of BRCA2 mutations in breast ovarian cancer families. Collectively 248 breast/ovarian cancer families have thus far been screened for BRCA2 mutations, and 17 have been identified (van der Looij *et al*, 2000, Grzybowska *et al*, 2000 and Górski unpublished data). Using Fishers exact test we observed a significant difference between the incidence of BRCA2 mutations in the breast/male stomach cancer families compared to the breast/ovarian cancer families (*P*<0.025).

## DISCUSSION

Currently there is little information about the nature and frequency of BRCA2 constitutional mutations in families selected for the coexistence of breast and stomach cancers. In an earlier study it was reported that male stomach cancer was over-represented in BRCA2 mutation positive families. Verification of this relationship between breast and stomach cancer in our series of cases suggests that this disease constellation can be used as a phenotypic indicator for the pre-selection of families for BRCA2 testing. Unfortunately, the stomach cancer patients had succumbed to their disease and it was impossible to obtain mutation data from any of them. Notwithstanding, the stomach cancer cases were on the same transmitting lineage of the mutation, were diagnosed with disease under the age of 55 and it remains highly likely that they were gene carriers.

The SIRs for breast and stomach cancer in the families presented in this report is highly significant, indicating that it is a real entity and not a chance association, and it provides additional evidence that male stomach cancer is part of the spectrum of disease in BRCA2 families. Similarly, other cancers are over-represented providing additional evidence that BRCA2 mutations result in a less restricted disease phenotype than BRCA1 mutations. A significant difference in cancer incidence was observed between the two groups, which could be accounted for by the increased frequency of breast cancer in the mutation negative group. This observation is most likely due to fragmentary pedigree information (due to the decimation of this population in recent history) and is unlikely to represent an actual difference between the two groups.

Three mutations in this study can be clearly identified as causative, whilst the remaining three represent missense changes which can not unequivocally be assigned as causative. The missense changes occurred in a small region of the BRCA2 gene (from position 6443 to 6612) within 169 base pairs of one another and were all identified in patients with breast cancer and not in 100 control subjects. The amino acid changes conferred by the missense changes were not conservative, one change was a tyrosine to cysteine, the second a serine to cysteine and the third a lysine to asparagine. The introduction of a cysteine is likely to interact with other cysteines, which occur within this region of the gene and thereby disrupt the structural and functional activity of the protein. Since we do not know of any functional domains in this region of the gene it is impossible to determine the functional significance of these changes. Nevertheless, the evidence suggests that these missense mutations are likely to affect the function of the protein.

It has been shown that BRCA2 mutations are characterised by a lower level of penetrance than in BRCA1 mutation carriers (Boyd, 1996; Levy-Lahad *et al*, 1997). The results presented here support these findings, since we detected BRCA2 changes almost twice as frequently in patients without cancers among first degree relatives compared to patients with cancers diagnosed in parents or siblings (Boyd, 1996; Roa *et al*, 1996; Levy-Lahad *et al*, 1997; Risch *et al*, 2001).

The frequency of BRCA2 mutations identified in this population compared to similar studies targeting breast/ovarian cancer families indicates that the stomach/breast constellation may be a more specific phenotypic marker for BRCA2 mutations, and as such, be of benefit in assigning patients for BRCA2 mutation screening.

Our results confirm and extend the notion that BRCA2 is likely to be the molecular basis of at least a subset of stomach cancer patients. The breast – stomach phenotype is one of several types of familial tumour aggregation at varying sites associated with BRCA2 gene mutations. BRCA2 changes have also been related to other aggregations of malignancy, which include predispositions to ovarian, pancreatic or skin tumours (BCLC, 1999).

Further investigations are needed in order to describe the actual involvement of BRCA2 mutations in different types of familial aggregation of cancers and to assess whether or not there are histopathological differences in stomach cancers that are associated with mutations in this gene.
